# Evaluation of Health Pattern and Stress Levels among Patients Undergoing Alcohol Addiction Treatment—A Cross Sectional Study

**DOI:** 10.3390/jcm12154959

**Published:** 2023-07-28

**Authors:** Mateusz Curyło, Marlena Rynkiewicz-Andryśkiewicz, Przemysław Andryśkiewicz, Marcin Mikos, Dariusz Lusina, Jan W. Raczkowski, Monika Pajewska, Olga Partyka, Katarzyna Sygit, Marian Sygit, Elżbieta Cipora, Mateusz Kaczmarski, Roman Załuska, Tomasz Banaś, Łukasz Strzępek, Edyta Krzych-Fałta, Ewa Bandurska, Weronika Ciećko, Michał Zabojszcz, Barbara Maciuszek-Bartkowska, Artur Kotwas, Anna Knyszyńska, Dariusz A. Kosior, Michał Marczak, Aleksandra Czerw, Remigiusz Kozłowski

**Affiliations:** 1 Department of Internal Medicine, Rehabilitation and Physical Medicine, Medical University of Lodz, 90-647 Lodz, Poland; 2 Medical Rehabilitation Department, The Ministry of the Interior and Administration Hospital, 30-053 Cracow, Poland; 3 Department of Treatment of Alcohol Abstinence Syndromes, Independent Public Healthcare Facility in Lezajsk, 37-300 Lezajsk, Poland; 4 Faculty of Medicine and Health Sciences, Andrzej Frycz Modrzewski Cracow University, 30-705 Cracow, Poland; 5 Department of Orthopedics and Traumatology, University Hospital in Cracow, 30-688 Cracow, Poland; 6 Department of Economic and System Analyses, National Institute of Public Health NIH-National Research Institute, 00-791 Warsaw, Poland; 7 Department of Health Economics and Medical Law, Medical University of Warsaw, 01-445 Warsaw, Poland; 8 Faculty of Health Sciences, Calisia University, 62-800 Kalisz, Poland; 9 Medical Institute, Jan Grodek State University in Sanok, 38-500 Sanok, Poland; 10 Department of Management and Logistics in Health Care, Medical University of Lodz, 90-131 Lodz, Poland; 11 Department of Radiotherapy, Maria Sklodowska-Curie Institute-Oncology Center, 31-115 Cracow, Poland; 12 Clinical Department of General and Oncological Surgery, Saint Raphael Hospital, 30-693 Cracow, Poland; 13 Department of Basic of Nursing, Faculty of Health Sciences, Medical University of Warsaw, 01-445 Warsaw, Poland; 14 Center for Competence Development, Integrated Care and e-Health, Medical University of Gdansk, 80-204 Gdansk, Poland; 15 Collegium Medicum, Jan Kochanowski University, 25-317 Kielce, Poland; 16 Department of Biophysics, Carol Marcinkowski Medical University in Poznan, 60-780 Poznan, Poland; 17 Independent Research and Biostatistics Laboratory, Department of Social Medicine, Pomeranian Medical University in Szczecin, 71-210 Szczecin, Poland; 18 Department of Humanities and Occupational Therapy, Pomeranian Medical University in Szczecin, 70-103 Szczecin, Poland; 19 Mossakowski Medical Research Institute, Polish Academy of Sciences, 02-106 Warsaw, Poland; 20 Collegium of Management, WSB University in Warsaw, 03-204 Warsaw, Poland

**Keywords:** alcohol, stress, coherence, health behavior, addiction

## Abstract

Alcohol consumption is linked to over 200 diseases and injuries. It is also classified as a risk factor for several types of neoplasms as well as infectious diseases (i.e., HIV and tuberculosis). In 2019, among people aged 25 to 49, alcohol use was the leading risk factor for attributable burden of disease. There are many factors that affect alcohol drinking patterns such as social and economic status, social norms, cultural customs, availability of alcohol, etc. Stress also plays a significant role in the process of developing alcohol addiction. The aim of our study was to examine health patterns and stress levels among patients undergoing alcohol addiction treatment. The study sample consisted of 104 patients who were treated in a hospital ward due to alcohol dependence. Three standardized questionnaire tools were used to measure the sense of coherence and the level of stress among those patients. The main results suggest that the level of perceived stress correlated negatively with all dimensions of sense of coherence and all indicators of health behaviors, however, age was positively correlated with positive mental attitude, proper eating habits, and health behaviors. In conclusion, it is worth noting that developing patterns for positive health behaviors will make it possible to avoid alcohol dependence or reinforce the treatment results if alcohol dependence syndrome occurs.

## 1. Introduction

The harmful use of alcohol is associated with three million deaths per year worldwide [1]. According to the data provided by Eurostat in 2021, among the European Union member states, the highest percentage of expenditure on alcoholic beverages was recorded in Latvia (5%), Estonia (4.7%), and Poland (3.7%) [2]. The lowest percentage, in turn, was recorded in Spain (1.4%), the Netherlands (1.3%), Greece (1%), and Italy (1%). It is estimated that in Poland, there are approximately 700 thousand people addicted to alcohol, which constitutes approximately 2% of the population [3]. According to the Eurostat data, 8.4% of the European Union population aged over 15 consume alcohol daily. The episodes of intense alcohol consumption, more than 60 g of pure ethanol on one occasion at least once a month, were recorded in the EU from 3.5% in Cyprus up to over 30% in Germany (30.4%), Luxembourg (34.3%), and Romania (35.0%), reaching the highest value of 37.8% in Denmark. In Poland, the percentage reached approximately 17% [3]. Alcohol dependence syndrome is a complex of physiological, behavioral, and cognitive phenomena, among which drinking alcohol dominates other behaviors that previously had a greater value to the patient. One of the main symptoms of the dependence syndrome is constituted by alcohol craving [4]. Alcohol dependence can also be defined as a condition characterized by an impaired ability to stop or control alcohol use despite adverse social, occupational, or health consequences [4]. However, dependence constitutes a complex health condition that also includes social, economic, and family-related aspects as well as the general health condition of the patient or the occurrence of comorbidities [5,6]. According to the current medical knowledge, a holistic approach should be adopted toward treating alcohol addiction, taking into consideration the overall health condition of patients [6].

There are multiple definitions of health behaviors. These can be defined as any activities undertaken by individuals in connection to their health or those that have a proven influence on health [7]. According to Gochman, health behaviors include elements such as convictions, expectations and predictions, motives, ways of thinking and emotional personality mechanisms as well as observable behavioral patterns connected with maintaining, strengthening, and restoring health [8]. Gochman’s definition constitutes the theoretical basis for the Health Behavior Inventory (HBI), serving as a tool that enables the measurement of general health behaviors [8]. Three groups of factors shaping health behaviors are distinguished (i.e., predisposing, enabling, and reinforcing). Predisposing resources are knowledge, attitudes, values; enabling resources are the access to benefits as well as their quality, while reinforcing resources are connected to living conditions and cultural and social standards [8]. In contrast to pro-health behaviors, unhealthy behaviors include risky driving, unsafe sexual behavior, smoking, excessive alcohol consumption, and the use of toxic and psychoactive substances [6]. Long-term drinking of large amounts of alcohol (over 40 mL per day) may contribute to the development of cardiovascular diseases, liver diseases, or cancer. Frequent binge drinking can lead to addiction, which can be damaging not only in terms of physical health, but also deteriorate the emotional and social well-being of a person, interfering with their work performance, family, and social relationships [8].

The literature includes evidence that proves the connection between stress and negative health behaviors [9,10]. Stress can be defined as an emotionally or physiologically overwhelming event that results in adaptive or maladaptive processes necessary to restore body homeostasis. Stress is a risk factor in addiction; it influences well-being and health behaviors [11]. Prolonged exposure to stress has been linked to increased alcohol consumption, which in turn is a manifestation of negative health behavior [11]. Numerous leading theories referring to the development of addictions emphasize the crucial role of stress in this process [10,12,13,14,15]. The link between stress and alcohol consumption has already been noted within the early stage of conducting studies devoted to alcohol consumption [16]. According to the tension reduction hypothesis, stress increases anxiety, and in response, alcohol is consumed in order to reduce its level. The link between stress and alcohol has additionally been connected to observations showing that in persons addicted to alcohol, physiological reactions to stress were disturbed [17]. Currently, epidemiological data confirm the connection between stress and disorders related to alcohol consumption. However, it is not a cause–effect relationship; in various circumstances, stress does not necessarily lead to alcohol consumption. Genetic factors and past experiences play an important role in this interaction. This shows a more complex relationship between feeling stressed and drinking alcohol, and further research is necessary on this subject [17,18].

The aim of the present paper was to assess health behaviors, the sense of coherence as well as the stress experienced by patients undergoing alcohol addiction treatment.

## 2. Materials and Methods

The sample consisted of 104 participants aged 25–70 (M = 42.78; SD = 10.57), 89 males aged 26–70 (M = 42.77; SD = 10.44) and 13 females aged 25–63 (M = 42.85; SD = 11.80) who were treated at the Department of Treatment of Alcohol Abstinence Syndromes, An Independent Public Healthcare Facility in Lezajsk, Poland. Two participants did not provide information about gender. The following tools presented below were used in the study. For the outcome variables, the following questionnaire was used:

(a) Health Behavior Inventory (HBI) by Zygfryd Juczyński—this allows for the assessment of the general indicator of the severity of health behaviors as well as the results relating to individual categories of these behaviors [19]. It contains 24 statements describing various types of health-related behaviors. Responses were assessed in four categories of health behavior issues: proper eating habits, prophylactic behaviors, positive mental attitude, and health practices. The results can be used to help program preventive actions, identify directions for behavior modification, and monitor changes in health practices. Alcohol addiction is a disease that affects the overall health behaviors, it weakens other areas of behavior. Theoretical validity was assessed by correlating the HBI results with the results in tests measuring variables that should be related to health behaviors. Cronbach’s α reliability coefficient for the entire Inventory was 0.85.

For the variables analyzed as predictors, the following questionnaires were used:

(b) The Orientation to Life Questionnaire (SOC-29)—the questionnaire consists of 29 items divided into three subscales made of components of the sense of coherence (SOC) according to Antonovsky: 11 questions checking the area of comprehensibility (ability to understand and integrate), 10 referring to the concept of manageability (ability to handle), and eight questions measuring the scales of meaningfulness (ability do make sense). These abilities allow patients to cope with disease. The higher the level in these areas, the greater the individual’s potential to successfully cope with a difficult life event or illness. In the case of people addicted to alcohol, it allows one to determine the level of resources needed to cope with the disease. The Cronbach’s α reliability coefficient of the SOC-29 research studies varied between 0.70 and 0.95 from 124 studies [20].
(c) The Perceived Stress Scale (PSS-10)—this questionnaire consists of 10 questions examining the subjective feelings related to personal problems, related behaviors, and ways of dealing with difficult situations. The overall score of the scale is the sum of all points, where the higher the score, the greater the intensity of stress experienced. According to studies, PSS-10 has a good internal consistency reliability with a Cronbach’s α reliability coefficient range between 0.75 and 0.91.

## 3. Results

### 3.1. Descriptive Statistics

Table 1 presents the descriptive statistics for the analyzed variables (i.e., mean values, standard deviations, minimum and maximum values, measures of skewness and kurtosis and Cronbach’s α reliability coefficients) in our study.

The values for the measures of skewness and kurtosis did not indicate substantial deviation from the normal distribution. Therefore, subsequent analyses were based on parametric statistical methods.

### 3.2. Correlation Analysis

Table 2 presents the values of the Pearson’s correlation coefficients between the analyzed variables including the participants’ age.

### 3.3. Correlation Coefficients between the Analyzed Variables

The level of perceived stress correlated negatively with all dimensions of sense of coherence and all indicators of health behaviors. All dimensions of sense of coherence correlated positively with positive mental attitude, correct eating habits, and health behaviors in general. Manageability (item example: “When you think of the difficulties you are likely to face in important aspects of your life, do you have the feeling that you will always succeed in overcoming the difficulties?”), meaningfulness (item example: “You anticipate that your personal life in the future will be full of meaning and purpose”), and general sense of coherence correlated positively with preventive behavior. Comprehensibility (reversed item example: “Has it happened in the past that you were surprised by the be-haviour of people whom you thought you knew well?”), manageability, and general sense of coherence correlated positively with health practices. The participants’ age correlated positively with positive mental attitude, correct eating habits, and health behaviors in general.

### 3.4. Perceived Stress and Sense of Coherence as Predictors of Health Behaviors

Perceived stress, sense of coherence, and the participants’ age were analyzed as predictors of health behaviors. Health behaviors were analyzed as outcome variables. Many statistically significant correlations between the analyzed variables were detected (see Table 2). Therefore, to avoid multicollinearity, the stepwise algorithm of regression analysis was applied. Each indicator of health behaviors was analyzed in a separate statistical model. The results are presented in Table 3.

Perceived stress and manageability were statistically significant predictors of positive mental attitude. The relationship between perceived stress and positive mental attitude was negative and the relationship between manageability and positive mental attitude was positive. The lower the level of perceived stress and the higher the level of manageability, the higher the level of positive mental attitude. However, the relationship between perceived stress and positive mental attitude was stronger. This explained 34.8% of positive mental attitude variance, while the relationship between manageability and positive mental attitude was weaker. When the level of perceived stress was controlled, the relationship between manageability and positive mental attitude explained 7.5% of the variance.

Meaningfulness and the participants’ age were predictors of preventive behavior. Both were positively related to the dependent variable. Therefore, the higher the level of meaningfulness and the older the participants, the higher the level of preventive behavior. The relationship between meaningfulness and preventive behavior was stronger than the relationship between the participants’ age and preventive behavior. The former explained 10.1% and the latter explained 5.6% of variance. It is also worth noticing that both meaningfulness and the participants’ age explained less of the preventive behavior level individual differences than perceived stress did alone regarding positive mental attitude. Perceived stress and the participants’ age were statistically significant predictors of correct eating habits. The relationship between perceived stress and correct eating habits was negative, while the relationship between the participants’ age and correct eating habits was positive. The higher the level of perceived stress, the lower the level of correct eating habits. The older the participants, the higher the level of correct eating habits. Perceived stress accounted for 18.2% of the correct eating habits’ variance, and the participants’ age accounted for 5.3%. Both accounted for 23.5% of the individual differences regarding correct eating habits. Perceived stress was the only predictor of health practices. It accounted for 4.8% of the health practices’ variance. The higher the level of perceived stress, the lower the level of health practices. Perceived stress and the participants’ age were statistically significant predictors of health behaviors in general. The relationship between perceived stress and health behaviors was negative, while the relationship between the participants’ age and health behaviors was positive. The higher the level of perceived stress, the lower the level of health behaviors. The older the participants, the higher the level of health behaviors. Perceived stress accounted for 23.9% of the health behaviors’ variance, and the participants’ age accounted for 5.5%. Both accounted for 29.4% of individual differences regarding health behaviors in general.

## 4. Discussion

Analyses conducted by the authors demonstrated that the level of perceived stress was negatively correlated with the health behavior indicators. Similar results were obtained in the study by Satre et al., which proved that alcohol abuse was connected to behavioral health threats such as smoking and negative eating habits [21]. Studies related to other aspects of life also indicate a positive relationship between the sense of coherence and preventive health behaviors. Individuals with high SOC level will more likely be involved in pro-health behaviors such as physical activity or lower alcohol consumption [22]. The sense of coherence demonstrates a strong link with health perception and quality of life as well as a direct influence on stress [23].

This study demonstrated that age was positively correlated with positive mental attitude, proper eating habits, and health behaviors. Similar results were obtained in the study by Deeks et al., which proved that participants aged over 51 would undergo screening tests more often [24]. This could be explained by the fact that the consolidation of positive patterns of health behavior contributes to the accumulation of good habits throughout life. Older participants would also have their annual health check-up performed more frequently than the younger ones. Positive relationships between health behaviors and mental health were also confirmed in the studies by Hautekiet et al. and Richardson et al. [25,26]. The results obtained by Nilsson et al. indicated the correlation between the sense of coherence and physical well-being. The authors discussed the mutual influences of these two factors [27]. High SOC level influences greater physical well-being resulting from choosing pro-health behaviors. Moreover, it is likely that in individuals manifesting positive health behaviors, a reinforcement of the sense of coherence will take place. Positive correlation between the sense of coherence and mental attitude was also indicated in the study by Nilsson. Higher sense of coherence has a positive influence on the better management of mental resources [27].

The analyses demonstrate that the sense of meaningfulness and age constituted predictors of preventive behaviors. In the studies by Fernandes et al. and Storeng et al., it was proven that preventive behaviors play a more significant role with age [23,24,28,29,30]. This correlation can probably be explained by the greater probability of positive health behaviors among elderly socially engaged individuals as well as by the fact that with age, taking care of health and preventive behaviors become increasingly important for patients [24].

## 5. Limitations

One of the main limitations of the presented paper was constituted by the cross-sectional study being performed, without later repetition and verification of measurements over time. Size of the study group as well as not being able to compare the results between patient clusters can also be considered as a limitation. However, the aim of the study was not to compare changes over time, but rather to assess the health behaviors among individuals addicted to alcohol undergoing treatment for their addiction.

Moreover, because the study was conducted when the participants were in the first stage of their treatment, activity of the defense mechanisms of addiction, especially the mechanism of illusion and denial, could affect the participants’ responses. Still, we believe that the results acquired can enhance the insight into the functioning of patients addicted at the beginning of their treatment.

## 6. Conclusions

Strengthening internal resources for managing difficult and stressful situations may contribute to enhanced results of alcohol addiction therapy. It is necessary to pursue the goal of developing constructive stress management tools among those addicted to alcohol during their therapy as well as within preventive measures among the population at risk. Developing patterns of healthy behaviors will make it possible to avoid alcohol dependence or reinforce treatment results if alcohol dependence syndrome occurs [31]. This requires further research on using health behavior resources as a stress management mechanism, particularly among those suffering from addiction.

## Figures and Tables

**Table 1:** Descriptive statistics for the analyzed variables HBI (Health Behavior Inventory).

Variables	M	SD	Min	Max	S	K	α
Perceived stress	21.79	6.92	4	38	−0.24	−0.29	0.87
Comprehensibility	42.20	9.97	16	63	−0.27	−0.44	0.78
Manageability	45.39	9.06	26	65	−0.05	−0.40	0.75
Meaningfulness	39.90	8.42	13	56	−0.69	0.41	0.79
Sense of coherence	127.50	23.13	63	176	−0.25	−0.30	0.89
Positive mental attitude	19.90	4.83	7	30	−0.23	−0.26	0.79
Preventive behavior	19.05	5.22	6	30	−0.02	−0.36	0.71
Correct eating habits	15.96	4.70	6	25	0.01	−0.74	0.75
Health practices	16.40	4.08	6	27	−0.07	−0.20	0.54
Health behaviors	71.32	14.69	30	107	0.08	−0.21	0.87

M—mean value; SD— standard deviation; min—minimum value; max—maximum value; S—measure of skewness; K—measure of kurtosis; α—Cronbach’s alpha reliability coefficient.

**Table 2:** Correlation coefficients between the analyzed variables.

Variables	1.	2.	3.	4.	5.	6.	7.	8.	9.	10.
1. Perceived stress	-	-	-	-	-	-	-	-	-	-
2. Comprehensibility	−0.576 **	-	-	-	-	-	-	-	-	-
3. Manageability	−0.548 **	0.666 **	-	-	-	-	-	-	-	-
4. Meaningfulness	−0.419 **	0.485 **	0.528 **	-	-	-	-	-	-	-
5. Sense of coherence	−0.616 **	0.869 **	0.871 **	0.780 **	-	-	-	-	-	-
6. Positive mental attitude	−0.588 **	0.398 **	0.545 **	0.438 **	0.544 **	-	-	-	-	-
7. Preventive behavior	−0.267 **	0.095	0.250 *	0.287 **	0.243 *	0.576 **	-	-	-	-
8. Correct eating habits	−0.444 **	0.255 **	0.314 **	0.202 *	0.307 **	0.482 **	0.563 **	-	-	-
9. Health practices	−0.292 **	0.217*	0.221 *	0.051	0.199 *	0.310 **	0.364 **	0.525 **	-	-
10. Health behaviors	−0.511 **	0.307 **	0.430 **	0.325 **	0.419 **	0.774 **	0.826 **	0.825 **	0.677 **	-
11. Age	−0.102	0.024	0.077	0.031	0.051	0.216 *	0.232 *	0.259 **	0.101	0.265 **

* *p* < 0.05; ** *p* < 0.01.

**Table 3:** Analysis of perceived stress and sense of coherence as predictors of health behaviors.

Dependent Variable	Predictors	B	t	*p*	ΔR2
Positive mental attitude	Perceived stress	−0.42	−4.50	0.001	0.36
	Manageability	−0.33	3.51	0.001	0.08
Preventive behavior	Meaningfulness	0.30	3.18	0.002	0.10
	Age	0.24	2.47	0.015	0.06
Correct eating habits	Perceived stress	−0.39	−4.28	0.001	0.18
	Age	0.23	2.55	0.012	0.05
Health practices	Perceived stress	−0.22	−2.19	0.031	0.05
Health behaviors	Perceived stress	−0.45	−5.15	0.001	0.24
	Age	0.24	2.69	0.008	0.06

B—the standardized regression coefficient; t—the value of test for a predictor’s significance; *p*—statistical significance; ΔR2—the change of determination coefficient when the predictor is added to a model.

## Data Availability

The original data collected to conduct this study can be made available from the authors.
